# Developmental Outcomes at 24 Months of Age in Toddlers Supplemented with Arachidonic Acid and Docosahexaenoic Acid: Results of a Double Blind Randomized, Controlled Trial

**DOI:** 10.3390/nu9090975

**Published:** 2017-09-06

**Authors:** Angela M. Devlin, Cecil M. Y. Chau, Roger Dyer, Julie Matheson, Deanna McCarthy, Karin Yurko-Mauro, Sheila M. Innis, Ruth E. Grunau

**Affiliations:** 1 Department of Pediatrics, University of British Columbia, BC Children’s Hospital Research Institute, A4-194 950 West 28th Ave, Vancouver, BC V5Z 4H4, Canada; cchau@bcchr.ca (C.M.Y.C.); radyer@mail.ubc.ca (R.D.); jmatheson@bcchr.ca (J.M.); rgrunau@bcchr.ca (R.E.G.); 2 DSM Nutritional Products, Columbia, MD 21045, USA; Deanna.McCarthy@dsm.com (D.M.); Karin.Yurko-Mauro@dsm.com (K.Y.-M.)

**Keywords:** toddlers, long chain polyunsaturated fatty acids, arachidonic acid, docosahexaenoic acid, neurodevelopment

## Abstract

Little is known about arachidonic acid (ARA) and docosahexaenoic acid (DHA) requirements in toddlers. A longitudinal, double blind, controlled trial in toddlers (*n* = 133) age 13.4 ± 0.9 months (mean ± standard deviation), randomized to receive a DHA (200 mg/day) and ARA (200 mg/day) supplement (supplement) or a corn oil supplement (control) until age 24 months determined effects on neurodevelopment. We found no effect of the supplement on the Bayley Scales of Infant and Toddler Development 3rd Edition (Bayley-III) cognitive and language composites and Beery–Buktenica Developmental Test of Visual–Motor Integration (Beery VMI) at age 24 months. Supplemented toddlers had higher RBC phosphatidylcholine (PC), phosphatidylethanolamine (PE), and plasma DHA and ARA compared to placebo toddlers at age 24 months. A positive relationship between RBC PE ARA and Bayley III Cognitive composite (4.55 (0.21–9.00), B (95% CI), *p* = 0.045) in supplemented boys, but not in control boys, was observed in models adjusted for baseline fatty acid, maternal non-verbal intelligence, and BMI z-score at age 24 months. A similar positive relationship between RBC PE ARA and Bayley III Language composite was observed for supplemented boys (11.52 (5.10–17.94), *p* < 0.001) and girls (11.19 (4.69–17.68), *p* = 0.001). These findings suggest that increasing the ARA status in toddlers is associated with better neurodevelopment at age 24 months.

## 1. Introduction

The *n*-6 and *n*-3 long chain polyunsaturated fatty acids, arachidonic acid (20:4*n*-6, ARA), and docosahexaenoic acid (22:6*n*-3, DHA), are found at high concentrations in the brain, predominantly as components of phospholipids, and have important roles in brain development [1,2,3]. Brain DHA accumulation in phosphatidylethanolamine (PE) and phosphatidylcholine (PC) begins during gestation and is estimated to continue postnatally into childhood [4].

Considerable emphasis has been placed on dietary ARA and DHA requirements for infants under one year of age. Arachidonic acid and DHA can be synthesized from the dietary essential fatty acids, linoleic acid (18:2*n*-6, LA) and alpha linolenic acid (18:3*n*-3, ALA), respectively. However, studies in animals have reported that DHA from maternal diet is a more efficient source of DHA for the developing fetal and infant brain compared to ALA in maternal diet [5,6]. Randomized trials of DHA and ARA supplementation have reported that infants fed with formula supplemented with DHA and ARA from the first week after birth have better visual acuity at 12 months of age than infants consuming formula with no DHA or ARA [7], better attention at four and nine months of age [8], and higher cognitive scores at 18 months of age [9]. The long-term benefits of the DHA and ARA supplementation have also been reported. The infants that were fed the formulas supplemented with DHA and ARA performed better in executive function, vocabulary, and intelligence at 3–5 years of age compared to the infants that were fed the formula with no DHA and ARA [10]. Other studies have also reported beneficial effects of DHA and ARA supplementation during infancy on indicators of cognitive function at six years of age [11].

Less is known about requirements of DHA and ARA during the period from 12 to 24 months of age. This time point is one of rapid neurological and physical development, but also a time of great transition in diet and well-known vulnerability to nutrient deficiencies. Human milk is the recommended and best sole source of nutrition for infants from birth to six months of age, and provides the infant with ARA and DHA. Current Canadian infant feeding guidelines state that whole (homogenized, full-fat) cows’ milk may be fed as a human milk alternate beginning at 9–12 months of age [12]. Cows’ milk is a rich source of protein and calcium, but is low in *n*-6 and *n*-3 fatty acids and has negligible ARA and DHA. One randomized, controlled trial reported that the median daily intake of DHA in toddlers (18–36 months of age) was 13.3 mg and that those who received a formula supplemented with DHA for 60 days had fewer upper respiratory tract infections compared to toddlers that received no DHA supplementation [13]. This suggests some potential benefits of dietary DHA in this age group. The objective of the current study is to examine the effects of ARA and DHA supplementation in toddlers from 12 to 24 months of age compared to children following their usual diet on cognitive, language and visual-motor development, and biomarkers of ARA and DHA status.

## 2. Materials and Methods

### 2.1. Subjects and Study Design

This was a prospective, longitudinal double blind, randomized placebo-controlled trial of DHA and ARA supplementation. Healthy term (37–41 weeks gestation) toddlers (*n* = 133) born in 2009–2013 were recruited through advertisements at community centers and local family events, and Vancouver Coastal Health immunization clinics in Vancouver, Canada at 12–14 months of age. Inclusion criteria at the time of enrollment included the following: healthy 12–14 month ±7 days toddlers; appropriate weight for gestational age at birth (2500–4000 g); singleton birth; maternal age 20–40 years at delivery; English as the primary language in the home; non-smoking home environment; currently breast-fed ≤2 times per day or fed ≤236 mL/day infant formula containing ARA and DHA; primary source of milk for the toddler was cow’s milk or other milk substitutes not containing ARA and DHA; and not received fish oil or other oil supplements and no intent to provide these during the duration of the study. Toddlers with known food allergies, metabolic, neurological, genetic, or immune disorders; and those that had been hospitalized for surgery, growth failure or any other event which was considered likely to impact the outcomes in this study were excluded. The recruitment and enrollment of toddlers in the study was completed under the direction of Dr. Sheila Innis and her staff at BC Children’s Hospital Research Institute from January 2010 to September 2014.

Toddlers were randomized and assigned without bias to receive the DHA/ARA supplement (200 mg/day DHA from DHASCO^®^-S oil, 200 mg/day ARA from ARASCO^®^ oil, DSM Nutritional Products) or control (400 mg/day corn oil), provided as sprinkles that were added to the toddlers’ food daily, from baseline until 24 months of age. Home visits were scheduled to provide the nutrition supplements and to collect study diaries on infant feeding and health. Our goal was to provide a nutrition supplement of DHA + ARA to maintain DHA and ARA nutrition equivalent to what a toddler would receive if the mother were to continue breast-feeding the toddler from 12 to 24 months of age. To avoid any possibility that participation in this study could lead to discontinuation of breast-feeding, only mothers and their toddlers in whom breast-feeding had stopped or were breast-feeding twice a day or less were enrolled. During the first week, the parents were contacted twice by phone to check for any problems and to answer questions. The parents were also contacted when the toddler was 15, 17, 19, 21 and 23 months of age to provide the nutrition supplements as needed and collect routine information on illnesses. Toddler assessments for development, growth and dietary intake occurred at baseline and when the infant was 18 and 24 months of age. The data collection sheets were kept in a locked cabinet in the Innis Lab at the BC Children’s Hospital Research Institute.

Informed consent was provided by the child’s parent/guardian for inclusion in the study before they participated. The study was conducted in accordance with the Declaration of Helsinki, and the protocol was approved by the Ethics Committee of the University of British Columbia Clinical Research Ethics Board and the Children’s and Women’s Health Centre of British Columbia Research Ethics Board (certificate number: H09-02028). Clinical Trial Registry: NCT01263912 at clinicaltrials.gov.

### 2.2. Supplements

The DHA is derived from DHASCO-S, an algal (*Schizochytrium* sp.) triglyceride oil, and the ARA is derived from ARASCO, a fungal (*Mortierella alpina*) triglyceride oil, and are regarded as safe for use in foods and supplements [14,15]. Details of the composition of the supplement and control are provided in Table 1.

The supplement and control were prepared in individual packages of sprinkles, about two grams each. Two packages were taken per day. The supplement provided 100 mg DHA and 100 mg ARA per package. In appearance, the sprinkles resembled skim milk powder and had a very faint odor similar to skim milk powder. The placebo sprinkles contained corn oil and no DHA or ARA. The amounts of saturated, monounsaturated and LA from the corn oil are insignificant relative to the usual intake of fat and these fatty acids in the usual diet.

The parents were instructed to give one full serving (one package) twice a day, at separate meals, preferably one in the morning and one in the afternoon. The parents were instructed to mix the entire contents of one package with milk, or food such as yogurt or cereal. Written instructions were provided and explained in-person at the beginning of the study. The parents were also instructed not to exceed two sachets a day; not to “catch-up” missed supplements on another day; and if the child spilled part of the milk, replacement supplements should not be given.

The parents were given a calendar for each month of the 12 months that they were in the study, with one line for each week, and each day having two check boxes for administering one for each of the two servings (packages) of sprinkles per day. The parents were asked to complete the calendar at the end of each day. The supplements and control were provided in a container to store in the fridge; containers for empty, unused or damaged packages were also provided. Empty supplement packages and unused supplements were kept and returned at the study visits and were used as a measure of compliance.

### 2.3. Randomization

Each subject was assigned a unique random code number system, with the codes held in opaque sealed envelopes. The random number subject code was used on all data collection forms and blood samples. If a toddler withdrew from the study, that toddler’s unique random number code was not reassigned. Once the consent form was reviewed and signed, the random number code was opened. The supplement and control were prepared in identical packages, two identical supplement and two identical control packages, giving four groups each identified with a letter code, W, X, Y or Z. Each subject random number code was linked to one of the four potential study groups, W, X, Y, or Z. All research staff involved with the toddlers or with analyses of the samples were blinded to the group codes. The identity of the four codes was held in four separate sealed envelopes, in a locked cabinet, and opened at the completion of the study.

### 2.4. Measures of Nutritional Status

Venous blood (~10 mL) was collected from each toddler at baseline, enrolment at 12–14 months of age (±1 weeks); and at 24 months (±2 weeks) of age. For analyses of fatty acids status, plasma was separated from red blood cells by centrifugation, the buffy coat removed, and the plasma stored at −70 °C until later analysis. The red blood cells (RBC) were washed with saline two times to remove contaminating plasma, and stored at −70 °C until further analyses. Plasma and RBC lipids were extracted and lipid classes separated by HPLC, quantified with an evaporative light scattering detector, and recovered using a fraction collector. Fatty acids in the fractions of interest were converted to methyl esters, separated, and quantified by gas liquid chromatography as described previously [5,16].

### 2.5. Dietary Assessments

Dietary history information was collected using a food frequency questionnaire (FFQ). The FFQ included a diet history at baseline, and at ages 18 and 24 months to capture duration of breast-feeding, age of introduction and type of formula, dairy milks, milk substitutes and weaning foods. The FFQ was administered by interview with the parent or primary caregiver and covered the infant’s intake for the previous 4 weeks. Information on the frequency with which a food was eaten, portion size, brand name, methods of preparation, and types of meat, fish, poultry, eggs, fish and seafood(s) were collected. Care was taken to guide parents to capture foods provided by daycares or given by other caregivers [17,18,19].

The 3-day food diary was completed at baseline, 18 months of age, and 24 months of age and included a written record of all beverages and foods consumed and vitamin and mineral supplements, and included questions on the infant’s eating behavior, with the same format at each age. Food records were analyzed using Food Processor Nutrition Software (ESHA Research, Salem, OR) and the Canadian Nutrient File (Health Canada, CNF version 2007b) and USDA Nutrient File. Total fat, LA, ALA, ARA, and DHA intakes were determined.

### 2.6. Child Outcome Measures

The Bayley Scales of Infant and Toddler Development 3rd Edition (Bayley-III) is a standardized test of infant development based on age-referenced norms [20]. At age 24 months, Cognitive and Language scales of the Bayley-III were administered. The Beery–Buktenica Developmental Test of Visual–Motor Integration (5th Ed.) (Beery VMI) was administered at age 24 months and involved the child copying geometric designs arranged in order of increasing complexity [21].

Measures of attention during play with age appropriate toys are widely used [22]. At age 18 and 24 months, single object free play (5 min), multiple object free play (5 min), and a distractibility task (3 min) were given, following the methods of Columbo and colleagues [22,23]. The child was positioned on the parent’s lap facing a table on which age appropriate toys were placed. Parents were asked to be quiet and avoid distracting or interacting with their child. The tester limited interaction to encouragement to play with the toy. Verbal communication with the child followed a standard script, and the sessions were timed with a stopwatch and recorded by a video camcorder, which also recorded elapsed time for detailed coding. In the distractibility task, show clips on a TV in the periphery of the play area were used as the distraction. All videos were viewed and analyzed by coders blinded to the infant’s study group and all other information about the child and family. Videos were coded using Observer XT 12 (Noldus Information Technology). For the single-object task and the multiple-objects task, mean duration and total duration of looking at the toy, total number of looks to the toy and total number of inattention episodes were measured. For the distractibility task percentage of duration the child turned away from the toy, latency to turn from the toy to the distractor, and the duration of looking at the distractor were calculated.

### 2.7. Maternal Intelligence

The Test of Nonverbal Intelligence (TONI-3rd Ed.), a language-free measure, was used to assess mother’s cognitive ability. The TONI-3 is a norm-referenced measure of intelligence, aptitude, abstract reasoning, and problem solving that requires no reading, writing, or speaking; subjects only have to point to indicate their response choice [24].

### 2.8. Statistical Analyses

Descriptive statistics were used to present baseline characteristics; group differences were determined by *t*-test for linear variables and by chi-squared test for categorical variables. Group differences in circulating *n*-6 and *n*-3 fatty acids and dietary intakes at baseline and age 24 months were determined by *t*-tests for linear variables and chi-squared test for categorical variables. The primary outcomes of the study were the Bayley-III Cognitive and Language composite scores and the Beery VMI. Differences in primary outcomes between supplement and control groups were determined by general linear models. Secondary outcomes of the study were the attention and distractibility scores. Generalized Linear Modeling (GZLM) and General Estimating Equations (GEE) were used to investigate the relationships between circulating ARA and DHA and primary outcomes.

## 3. Results

### 3.1. Baseline Characteristics of Subjects

**Figure 1:**
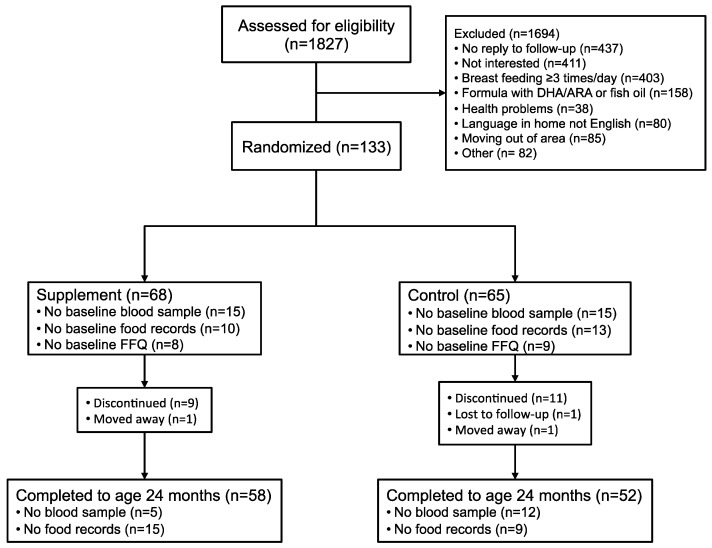
Flow chart illustrating the study subjects in the supplement and control group.

A total of 133 toddlers were enrolled into the trial; *n* = 68 in the supplement group and *n* = 65 in the control group (Figure 1). Of these, 82.7% (*n* = 110) completed the trial. The dropout rate was 14.7% (*n* = 10) in the supplement group and 20.0% (*n* = 13) in the control group. There were no differences in baseline characteristics between subjects that completed the study and those that dropped out of the study.

The baseline characteristics of the subjects are given in Table 2. All toddlers were healthy term-born babies with appropriate for gestational age birth weight. The majority of the toddlers were of European or Asian descent. There were no significant differences in the number of male subjects, gestational age, birth weight, age at baseline, zBMI at baseline and age 24 months, ethnicity, family income, maternal age at delivery, maternal education, and maternal nonverbal intelligence score (TONI-3) between the supplement group and the control group (Table 2).

### 3.2. Dietary Intakes at Baseline and Age 24 Months

At baseline, the supplement group had a greater percentage of toddlers (91.7%) that consumed fish compared to the control group (76.8%) (Table 3). There were no differences at baseline between the groups in the percentage of toddlers consuming eggs, poultry, or human milk. The supplement group also had a greater intake of ARA at baseline than the control group (Table 3). There were no differences in the intake of total dietary fat, LA, ALA, EPA or DHA. At age 24 months, there were no differences in dietary intake of any nutrients or food items.

### 3.3. Effect of the Supplement on Developmental Outcomes

The Bayley-III Cognitive and Language composite and Beery VMI scores at age 24 months showed no significant differences between the supplement and control groups in unadjusted models and models adjusted for baseline dietary ARA intakes (Table 4). Further separate analyses in girls and boys showed no effect of the supplement on developmental tests.

Inter-rater reliability for the attention tasks was assessed for look duration and episodes of attention on each task at each age (18 and 24 months) following Colombo et al. [22] using Cohen’s Kappa on 25% of the coding, rather than percent agreement. Mean Kappa for the single-object task was: 0.93 at age 18 months and 0.88 at age 24 months, for the multi-object task 0.99 at age 18 months and 0.99 at age 24 months, and for distractibility was 0.94 at age 18 months and 0.94 at age 24 months. There were no significant differences on the single-object, multiple-object or distractibility tasks between the groups at 18 or 24 months (Table S1). We further used GEE to examine relationships between inattention at 18 months and 24 months of age in the multiple toys task by supplementation group and sex, adjusting for zBMI at birth, child age at first visit, and mother non-verbal intelligence (TONI-3) as covariates. In these models, we found that boys receiving the supplement had significantly fewer inattention episodes than boys receiving the control at 24 months of age (*p* = 0.042).

### 3.4. Circulating n-3 and n-6 Fatty Acid Status and Developmental Outcomes

We quantified the circulating status of LA, ALA, ARA, eicosapentaenoic acid (EPA, 20:5*n*-3), and DHA in plasma and RBC PC and PE in the toddlers at baseline and at age 24 months. Baseline RBC PE and PC ARA were higher in toddlers in the supplement group compared to those in the control group (Table 5). There were no differences at baseline in circulating levels of LA, ALA, EPA, and DHA in toddlers from the supplement group compared to those in the control group. As expected, at age 24 months, the supplement group had a greater percentage of ARA and DHA in plasma and RBC PE and PC, and in RBC PE EPA compared to the control group, after Bonferroni adjustment for multiple comparisons (Table 5). This was accompanied by a lower percentage of LA, oleic acid (18:1*n*-9) in RBC PE and plasma, and a higher percentage of stearic acid (18:0) in RBC PC in the supplement group compared to the control group at age 24 months (Table S2).

**Figure 2:**
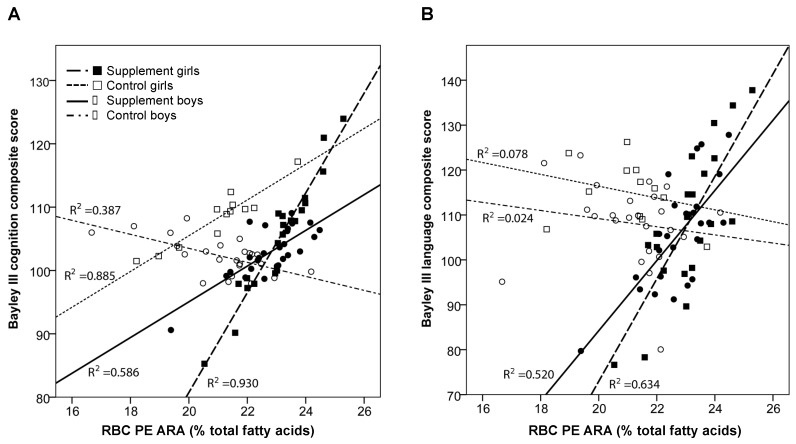
Relationship between RBC PE arachidonic acid (ARA) level and: (**A**) Bayley III cognitive composite scores; and (**B**) Bayley III language composite scores at age 24 months by supplement group and sex (supplement-treated girls (■); control-treated girls (□); supplement-treated boys (●); and control-treated boys (○).

We further examined the relationships between circulating ARA, EPA, and DHA levels, and the Bayley-III Cognitive and Language composite. Separate GZLM models examined relationships between RBC PC and PE and plasma *n*-6 and *n*-3 fatty acids at age 24 months and the Bayley-III Cognitive and Language composite. Interactions between treatment group (supplement, control) and sex were examined, with adjustments for zBMI at age 24 months, fatty acid levels at baseline, and maternal nonverbal intelligence score (TONI-3). The overall omnibus test was significant (χ^2^ = 18.23, *p* = 0.033). There was a significant interaction between supplement group, sex, and ARA in RBC PE (*p* = 0.019, Wald χ^2^ = 9.96, Table 6). Interactions are illustrated in Figure 2A. Contrasts were performed to further understand the significant 3-way interaction. There was a significant contrast between boys in the supplement group compared to the control group (*p* = 0.045); boys in the supplement group had higher Bayley-III Cognitive scores than boys in the control group at age 24 months (Table 7; Figure 2A) in models adjusted for baseline fatty acid level, maternal non-verbal intelligence score (TONI), and zBMI at age 24 months. There was no significant difference between supplement and control groups for girls (*p* = 0.092) (Table 7).

The same GZLM models were used to assess Bayley-III language composite. The overall omnibus was significant (Omnibus χ^2^ = 36.13, *p* ≤ 0.001). We observed a similar interaction between the supplement group, RBC PE ARA and sex (*p* = 0.004, Wald χ^2^ = 13.11, Table 6), as shown in Figure 2B. Both boys (*p* = 0.0004) and girls (*p* = 0.003) who received the supplement had higher Bayley-III Language composite compared to children in the control group.

## 4. Discussion

The objective of this prospective, longitudinal double blind, randomized controlled trial was to determine effects of supplementing toddlers with DHA and ARA from age 12 to 24 months on neurodevelopmental outcomes and biomarkers of ARA and DHA status. We found no differences between supplement and control-treated toddlers on BAYLEY-III cognition and language composite scores [20] and the Beery VMI [21], the primary outcomes of the study. As expected, the supplement increased circulating biomarkers of DHA and ARA status in RBC PE and PC and plasma from the toddlers. After adjusting for baseline fatty acid, zBMI at age 24 months, and mother’s nonverbal IQ (TONI-3), we found a positive association between ARA status in RBC PE, the largest fraction of circulating ARA, and cognition but only for boys in the supplement group. No relationships were observed for girls. Similarly, RBC PE ARA was positively associated with language scores for both boys and girls in the supplement group but not for those in the control group. In contrast, no relationships were observed between supplement group, DHA status, and neurodevelopmental outcomes. Overall, these findings suggest that increasing the status of ARA in RBC PE in infants taking a supplement containing ARA (200 mg/day) and DHA (200 mg/day) from 12–24 months of age is associated with better cognition and language.

Interestingly, we found a positive relationship between RBC PE ARA and neurodevelopmental outcomes but only in supplemented toddlers. This suggests that increasing the status of ARA to levels observed in the supplemented toddlers has positive effects on neurodevelopment. The biological relevance of this relationship is not known and requires further investigation. A small trial in preterm infants (*n* = 45) reported better psychomotor development at age 24 months in infants that were fed a formula with 0.67% ARA/0.33% DHA compared to those fed a formula with 0.37% ARA/0.37% DHA suggesting a positive effect of higher ARA in the formula [25]. Similar findings of a positive effect of ARA and DHA on neurodevelopment of preterm infants were reported in the infants at age six months [26]. Arachidonic acid and PE are important during development; they are critical components of neural membranes [27] and are required for the synthesis of endocannabinoids, especially anandamide and 2-arachidonylglycerol [28]. The endocannabinoids play important roles in neurotransmission and behavior, functioning through the cannabinoid (CB) receptor, CB1, in the brain. A study in adult male rats reported higher levels of anandamide and 2-arachidonoylglycerol in the brain of rats supplemented with ARA compared to rats fed a palm oil diet despite no changes in brain phospholipid ARA [29]. It seems reasonable to predict that supplementing the infants with DHA and ARA may affect tissue levels of endocannibinoids, especially in the brain, and mediate the relationship between RBC PE ARA and neurodevelopmental scores that we observed in the supplemented infants.

Additional evidence supporting the importance of ARA during development has recently been described in a mouse model. Studies in male mice deficient in delta-6 desaturase (D6D, *Fads2* -/- mice), the enzyme required for the desaturation of LA and ALA to ARA and DHA, respectively, reported that D6D deficient mice were smaller, had reduced levels of motor activity and coordination, and reduced ARA and DHA levels in RBC and brain compared to wild-type mice [30]. Interestingly, supplementing the D6D deficient mice with ARA containing formula during the suckling period improved growth and motor activity but not motor coordination [30]. Beneficial effects on growth and motor coordination were also reported in the D6D deficient mice supplemented with DHA; but DHA supplementation did not improve motor activity [31]. Interestingly, D6D deficient mice supplemented with both ARA and DHA showed the greatest improvements in motor coordination compared to unsupplemented D6D deficient mice or those supplemented with just ARA or DHA [31]. However, in contrast to these studies, questions regarding the requirement for dietary ARA during infancy have been raised and the subject of some current reviews [3,32]. Further, a meta-analysis of randomized trials of long chain *n*-3 and *n*-6 polyunsaturated fatty acid supplementation of term infants reported no effects on growth, regardless of whether the formula contained ARA or not [33].

In contrast to what we predicted, this trial observed no effect of increasing DHA status in toddlers between ages 12 and 24 months on neurodevelopmental outcomes. The DHA levels in RBC phospholipids and plasma from the toddlers in the supplement group on average increased 30–50% from baseline to 24 months of age, whereas, in the control group, the levels of DHA slightly decreased between baseline and the end of the study, clearly demonstrating effects of the supplement on DHA status. This is in line with a small study (*n* = 86) that reported increases in DHA status of toddlers supplemented with either 43 mg/day DHA or 130 mg/day DHA for 60 days at 18–24 months of age [13]; fewer respiratory illnesses were also reported in the infants receiving 130 mg/day DHA compared to those receiving no DHA supplementation.

Little is known regarding the nutritional status of long chain polyunsaturated fatty acids and optimal intakes for toddlers between ages 12 and 24 months. The optimal levels of dietary intakes of DHA and ARA to support neurological development during this time period are not known. Most studies to date have focused on effects of prenatal (maternal) supplementation or supplementation during the first year after birth. Findings of these studies have been variable. A systematic review of 11 randomized controlled trials of 5272 participants found no effect of maternal DHA supplementation during pregnancy or during pregnancy and lactation on neurocognitive outcomes, except for a positive effect of DHA supplementation on IQ scores in children at 2–5 years of age [34].

Beneficial effects of DHA and ARA supplementation of infants during the first year of life on neurodevelopmental outcomes have been reported. The DIAMOND study was a multicenter, double-blind, randomized controlled trial (*n* = 244) conducted in Kansas City and Dallas that compared infants that were fed a formula with no ARA and DHA with infants fed formulas supplemented with ARA (34 mg/100 kcal) and DHA, at three different levels (17 mg/100 kcal, 34 mg/100 kcal, and 51 mg/100 kcal), from the first week of life to 12 months of age. The trial reported better visual acuity at 12 months of age in infants fed the DHA and ARA supplemented formulas compared to infants fed the formula with no ARA or DHA [7]. Further secondary analyses of infants in the trial reported that those fed the DHA supplemented formulas had better attention at four, six and nine months of age (*n* = 122) [8]. Follow-up studies reported no effect of the DHA/ARA supplementation during the first year of life on neurodevelopmental outcomes at 18 months of age in infants from Dallas (*n* = 117) [9] and in infants from Kansas City (*n* = 81) [8]. However, the children that received the DHA/ARA supplemented formula in infancy at the Kansas City site performed better on neurodevelopmental tests in a long term follow up study at 3–5 years of age (*n* = 81) [10] and showed differences in brain electrophysiology at 6 years of age (*n* = 69) [35]. Similarly, a multicenter European trial of DHA and ARA supplementation of infants (*n* = 147) during the first four months of life reported no effects of the supplementation on IQ at six years of age but did report that the supplemented children were faster at processing information than the unsupplemented children [11]. Together, these findings suggest long-term neurodevelopmental effects of supplementing infants in the first year of life with DHA and ARA. This raises question as to whether there may be, as yet unidentified, long-term benefits at later time points during childhood of supplementing toddlers with DHA and ARA between ages 12 and 24 months.

The supplement provided 200 mg/day of ARA and DHA, which is approximately six times higher than the levels found in the diet of the toddlers at baseline and at age 24 months. In addition to the ARA/DHA consumed from breast milk or formula during the first year of life, a large number of the toddlers in the study (>75%) consumed DHA and ARA containing foods, including fish, eggs, and poultry, at baseline and this number increased to >80% of the toddlers consuming these foods at age 24 months. The question remains as to what the optimal dietary intake levels are for children at this age and what are the best indicators of status and function. Providing 200 mg/day of DHA increased circulating biomarkers on average by 29–50% but this was not associated with any differences on indicators of neurodevelopment. In contrast, the 200 mg/day each of ARA and DHA, increased circulating RBC PE ARA by only 6% and this was associated with neurodevelopment outcomes. In contrast, the RBC PC ARA and DHA increased by 38% and 50%, respectively, and plasma ARA and DHA increased by 43% and 54%, respectively, but we found no relationships between these biomarkers of ARA and DHA status and neurodevelopment. It is possible that the neurodevelopmental tests used in this trial were not sensitive to changes in DHA status but that other functional endpoints might be affected. As mentioned above, no effect of supplementing infants with DHA/ARA during the first year of life on the Bayley (version 2) scores at age 18 months of age were reported by Columbo et al. [10]. Interestingly, maternal DHA levels have been associated with better infant attention at 12 and 18 months [22,23]. In the present study, we found that after adjusting for baseline fatty acid, zBMI at age 24 months, and mother’s non-verbal IQ, boys in the supplementation group showed less inattention at age 24 months. Further research is needed to identify what markers of brain development and cognition are the best indicators of DHA and ARA function in toddlers during the age of 12–24 months.

Some limitations to the study must be considered when interpreting our results. Most importantly is whether the neurodevelopmental tests that were used in the trial, the Bayley-III, Beery VMI and attention tasks, are sensitive enough to detect effects of the DHA and ARA supplement on neurodevelopment. The sensitivity and limitations of these tests and appropriateness for nutritional interventions in healthy children have been raised [36]. Another limitation is the reliance on circulating concentrations of DHA and ARA as biomarkers of status. We have no other choice given we are studying young children. However, we do not know whether the circulating concentrations of fatty acids directly reflect levels that are found in tissues like the brain. It is reasonable to predict that uptake, incorporation, and use of fatty acids by tissues, is tissue and cell specific, and may not always reflect what is observed in circulation. We also cannot account for ARA and DHA stores that accrued in the toddlers prenatally and during the first year of life. There may also be genetic determinants of DHA and ARA metabolism that influence response of the infants to the DHA and ARA supplement. Prior studies have focused predominantly on variants in genes encoding enzymes required for the elongation and desaturation of essential fatty acids [37] but variants in other proteins governing the functional effects of DHA and ARA also warrant investigation. The sample size of the trial is also smaller than planned. The initial goal of the trial was to recruit 200 toddlers but enrollment of toddlers in the trial was slower than anticipated because of barriers relating to toddlers not meeting the inclusion criteria, such as breast-feeding <2 times per day; being fed <236 mL/day infant formula containing ARA and DHA; or taking fish oil or other oil supplements.

## 5. Conclusions

In summary, findings from this longitudinal, double-blind, randomized controlled trial of DHA and ARA supplementation of toddlers between 12 and 24 months of age found no effect on neurodevelopment. However, the study is limited because the cognitive tests that were used may not be sensitive enough to detect effects of DHA and ARA supplementation during this developmental period. A positive relationship between the status of ARA in RBC PE, in toddlers taking the supplement containing ARA (200 mg/day) and DHA (200 mg/day), and better neurodevelopment was observed. Further studies are required to determine the long-term effect of the supplement at later time points in childhood using more sensitive indicators of cognition.

## Figures and Tables

**Table 1:** Composition of supplements.

	Supplement (mg/Package)	Control (mg/Package)
Fish Gelatin	325–460	325–460
Sucrose	325–460	325–460
Corn Starch	310–440	310–440
Sodium Ascorbate	75–105	75–105
DHA	100	0
ARA	100	0
Corn Oil	0	445–625

Abbreviations: ARA, arachidonic acid; DHA, docosahexaenoic acid; the source of DHA and ARA was DHASCO-S, algal (*Schizochytrium* sp.) triglyceride oil; ARASCO, fungal (*Mortierella alpina*) triglyceride oil.

**Table 2:** Participant characteristics at baseline.

	Supplement	Control	*p*
Male sex, *n* (%)	40 (59)	41 (63)	0.618
Gestational age, weeks	39.7 (1.4)	39.6 (1.2)	0.992
Birth weight, g	3396 (508)	3471 (440)	0.422
Child age at baseline, months	13.3 (0.9)	13.5 (0.8)	0.146
zBMI, baseline	0.6 (1.0)	0.5 (0.9)	0.958
zBMI, 24 months	0.9 (0.9)	0.9 (0.9)	0.898
Ethnicity			
Asian	19 (27.9)	14 (21.5)	0.273
European	43 (63.2)	41 (63.1)	
First Nations	0 (0)	2 (3.1)	
Missing	6 (8.8)	8 (12.3)	
Family Income, *n* (%)			
<$30,000/year	3 (5.1)	9 (17.6)	0.079
$30,000/year–$50,000/year	8 (13.6)	5 (9.8)	
>$50,000/year	46 (62.7)	32 (62.7)	
Not indicated	2 (3.4)	5 (9.8)	
Maternal age at delivery, years	32.2 (4.3)	32.6 (4.9)	0.606
Maternal Education, *n* (%)			
Did not finish high school	1 (1.5)	0 (0)	0.810
High school	3 (4.4)	3 (4.6)	
College/vocational diploma	11 (16.2)	10 (15.4)	
University undergraduate degree	20 (29.4)	22 (32.3)	
University graduate/professional degree	27 (39.7)	21 (86.2)	
Missing	6 (8.8)	9 (13.8)	
Maternal Nonverbal Intelligence (TONI-3)	110.2 (16.2)	112.9 (14.6)	0.404

Abbreviations: zBMI, BMI standardized for age and sex. Data presented as mean (standard deviation), unless otherwise stated.

**Table 3:** Daily dietary intakes of children at baseline and age 24 months.

	Baseline				Age 24 Months		
	Supplement	Control	*p*		Supplement	Control	*p*
Total Fat, g	38.1 (12.7)	37.7 (11.9)	0.860	Total Fat, g	43.2 (12.7)	42.2 (14.4)	0.731
LA (18:2*n*-6), g	4.15 (2.0)	3.71 (1.5)	0.194	LA (18:2*n*-6), g	5.07 (2.3)	5.31 (2.9)	0.672
ALA (18:3*n*-3), g	0.59 (0.3)	0.54 (0.3)	0.386	ALA (18:3*n*-3), g	0.78 (0.5)	0.69 (0.4)	0.384
ARA (20:4*n*-6), mg	42.4 (35.4)	30.6 (22.0)	0.041	ARA (20:4*n*-6), mg	55.3 (40.0)	50.0 (32.5)	0.496
EPA (20:5*n*-3), mg	15.7 (37.5)	16.3 (47.3)	0.936	EPA (20:5*n*-3), mg	27.4 (67.1)	44.2 (77.8)	0.288
DHA (22:6*n*-3), mg	31.4 (51.8)	30.8 (66.1)	0.957	DHA (22:6*n*-3), mg	53.3 (98.9)	76.3 (115)	0.321
Fish in diet, *n* (%)	55 (91.7)	43 (76.8)	0.027	Fish in diet, *n* (%)	52 (89.7)	43 (82.7)	0.288
Egg in diet, *n* (%)	57 (95.0)	50 (89.3)	0.250	Eggs in diet, *n* (%)	54 (94.7)	47 (90.4)	0.384
Poultry in diet, *n* (%)	55 (91.7)	50 (90.9)	0.885	Poultry in diet, *n* (%)	54 (93.1)	47 (90.4)	0.603
Human milk, *n* (%)	13 (25.5%)	15 (25.9)	0.965	Human milk, *n* (%)	2 (4.7%)	1 (2.3)	0.557

Abbreviations: ALA, linolenic acid; ARA, arachidonic acid; DHA, docosahexaenoic acid; EPA, eicosapentaenoic acid; LA, linoleic acid. Data presented as mean (standard deviation), unless otherwise stated. Between group differences determined by *t*-tests for linear variables and chi-squared tests for categorical variables.

**Table 4:** Effect of ARA and DHA supplementation on developmental scores at age 24 months.

	Supplement	Control	Model 1	Model 2
Bayley-III Cognition Composite	103.9 (13.6)	103.7 (13.9)	*p* = 0.932	*p* = 0.723
Bayley-III Language Composite	105.6 (21.0)	106.9 (30.4)	*p* = 0.742	*p* = 0.648
Beery VMI	92.6 (21.2)	93.4 (11.0)	*p* = 0.748	*p* = 0.471

Abbreviations: ARA, arachidonic acid; Bayley-III, Bayley Scales of Infant and Toddler Development 3rd Edition; Beery VMI, Beery–Buktenica Developmental Test of Visual–Motor Integration (5th Ed.); DHA, docosahexaenoic acid.Data presented as mean (standard deviation). Between group comparisons by general linear models. Model 1, unadjusted. Model 2, adjusted for baseline dietary arachidonic acid intakes.

**Table 5:** Circulating *n*-3 and *n*-6 fatty acids levels of children at baseline and age 24 months.

Fatty Acid	Baseline			Fatty Acid	Age 24 Months		
	Supplement	Control	*p*		Supplement	Control	*p*
Linoleic acid	Percent fatty acids	Percent fatty acids		Linoleic acid	Percent fatty acids	Percent fatty acids	
RBC PE	5.2 (0.8)	5.1 (0.9)	0.562	RBC PE	3.8 (0.8)	5.4 (1.0)	<0.001
RBC PC	20.0 (1.6)	20.3 (2.1)	0.370	RBC PC	17.5 (2.1)	20.9 (2.4)	<0.001
Plasma	22.2 (2.5)	23.2 (2.4)	0.058	Plasma	19.1 (3.3)	23.3 (2.6)	<0.001
Arachidonic acid				Arachidonic acid			
RBC PE	21.7 (1.0)	20.9 (1.6)	0.008	RBC PE	23.0 (1.1)	21.0 (1.5)	<0.001
RBC PC	6.0 (1.1)	5.6 (0.9)	0.024	RBC PC	8.3 (1.8)	5.5 (0.8)	<0.001
Plasma	8.2 (2.3)	7.9 (1.5)	0.448	Plasma	11.7 (2.7)	7.8 (1.3)	<0.001
α-Linolenic acid				Linolenic acid			
RBC PE	0.14 (0.04)	0.13 (0.04)	0.380	RBC PE	0.12 (0.04)	0.15 (0.04)	<0.001
RBC PC	0.21 (0.06)	0.19 (0.06)	0.184	RBC PC	0.20 (0.06)	0.21 (0.06)	0.222
Plasma	0.26 (0.09)	0.25 (0.09)	0.586	Plasma	0.22 (0.07)	0.23 (0.07)	0.589
Eicosapentaenoic Acid				Eicosapentaenoic Acid			
RBC PE	1.1 (0.5)	1.1(0.7)	0.531	RBC PE	0.9 (0.3)	1.1 (0.5)	0.001
RBC PC	0.4 (0.2)	0.4 (0.3)	0.502	RBC PC	0.4 (0.1)	0.4 (0.2)	0.294
Plasma	0.6 (0.4)	0.6 (0.4)	0.533	Plasma	0.6 (0.3)	0.5 (0.3)	0.692
Docosahexaenoic Acid				Docosahexaenoic Acid			
RBC PE	7.5 (1.7)	7.6 (2.0)	0.750	RBC PE	9.7 (1.6)	6.5 (1.8)	<0.001
RBC PC	2.0 (0.6)	1.9 (0.6)	0.823	RBC PC	3.0 (0.8)	1.7 (0.6)	<0.001
Plasma	2.8 (1.1)	2.7 (0.9)	0.660	Plasma	4.3 (1.3)	2.6 (0.9)	<0.001

Abbreviations: PC, phosphatidylcholine; PE, phosphatidylethanolamine. Data presented as mean (standard deviation). Between group differences determined by *t*-tests and Bonferroni correction for multiple comparison (*p* < 0.0028).

**Table 6:** Relationships between circulating ARA and DHA with developmental scores at age 24 months.

	Supplement Group × Sex × Fatty Acid Interaction		
	Bayley-III Cognitive	Bayley-III Language	Beery VMI
ARA (20:4*n*-6)			
RBC PE	9.96 (0.019)	13.11 (0.004)	NS
RBC PC	NS	4.75 (0.191)	NS
Plasma	NS	1.39 (0.707)	NS
DHA (22:6*n*-3)			
RBC PE	NS	2.43 (0.488)	NS
RBC PC	NS	1.35 (0.717)	NS
Plasma	NS	0.607 (0.895)	NS

Abbreviations: ARA, archidonic acid; DHA, docosahexaenoic acid; PC, phosphatidylcholine; PE, phosphatidylethanolamine. Relationships determined by separate general linear models adjusted for sex, zBMI at 24 months of age, baseline fatty acid, and maternal non-verbal intelligence (TONI-3). Supplement × sex × fatty acid interaction. Data presented as B (*p*-value).

**Table 7:** Relationships between RBC phosphatidylethanolamine arachidonic acid levels and Bayley-III developmental outcomes in boys and girls at age 24 months.

	Bayley-III Cognitive		Bayley-III Language	
	B (95% CI)	*p*	B (95% CI)	*p*
**Boys**				
RBC PE ARA × Supplement	4.55 (0.12–9.00)	0.045	11.52 (5.10–17.94)	0.0004
**Girls**				
RBC PE ARA × Supplement	3.86 (−0.64–8.350)	0.092	11.19 (4.69–17.68)	0.003

Abbreviations: ARA, archidonic acid; PE, phosphatidylethanolamine. Relationships determined by separate general linear models adjusted for zBMI at age 24 months, baseline fatty acid, and maternal non-verbal intelligence (TONI-3). Supplement × sex.

## Supplementary Materials

The following are available online at www.mdpi.com/2072-6643/9/9/975/s1. Table S1, Effect of ARA and DHA supplement on attention and distractibility measures at age 18 and 24 months; Table S2, Major fatty acids levels of children at baseline and age 24 months.
