# Supplementary material for: Developmental Outcomes at 24 Months of Age in Toddlers Supplemented with Arachidonic Acid and Docosahexaenoic Acid: Results of a Double Blind Randomized, Controlled Trial
Source: Nutrients. 2017 Sep 6;9(9):975. doi: 10.3390/nu9090975 (PMC5622735; doi:10.3390/nu9090975)
Supplement: Supplementary file 1 [file nutrients-09-00975-s001.zip › nutrients-216615-supplementary.pdf]

Measures of attention: The child was positioned on the parent's lap facing a table on which age appropriate toys were placed. Parents were asked to be quiet and avoid distracting or interacting with their child. The tester limited interaction to encouragement to play with the toy. Verbal communication with the child followed a standard script, and the sessions were timed with a stopwatch and recorded by a video camcorder, which also recorded elapsed time for detailed coding. In the distractibility task, show clips on a TV in the periphery of the play area were used as the distraction. All videos were viewed and analyzed by coders blinded to the infant`s study group and all other information about the child and family. Videos were coded using Observer XT 12 (Noldus Information Technology). For the single-object task and the multiple-objects task, mean duration and total duration of looking at the toy, total number of looks to the toy and total number of inattention episodes were measured. For the distractibility task percentage of duration the child turned away from the toy, latency to turn from the toy to the distractor, and the duration of looking at the distractor were calculated.

Table S1. Effect of ARA and DHA supplement on attention and distractibility measures at age 18 and 24 months

	Supplement	Placebo	*p*
**18 months of age**			
Single object mean duration at toy, seconds	16.9 (10.5)	17.9 (9.8)	0.619
Single object total duration at toy, seconds	222.9 (56.7)	239.7 (56.3)	0.158
Single object total looks at toy, counts	16.0 (6.5)	15.4 (5.8)	0.633
Single object total inattention episode, count	14.5 (6.4)	14.4 (5.8)	0.930
Multiple objects mean duration at toy, seconds	225.9 (63.5)	240.2 (44.1)	0.209
Multiple objects total duration at toy, seconds	24.4 (15.0)	24.6 (16.4)	0.944
Multiple objects total looks at toy, counts	11.2 (5.4)	12.2 (4.7)	0.392
Multiple objects total inattention episode, counts	8.5 (5.4)	9.8 (4.9)	0.227
Distractibility total duration TV/total duration toy	36.3 (19.5)	34.8 (16.4)	0.730
Distractibility latency, seconds	14.0 (8.3)	15.5 (10.2)	0.509
Distractibility total looks at TV, counts	12.8 (3.3)	12.5 (3.7)	0.725
**24 months of age**			
Single object mean duration at toy, seconds	23.7 (15.6)	25.7 (17.4)	0.571
Single object total duration at toy, seconds	236.6 (55.9)	248.0 (45.5)	0.304
Single object total looks at toy, counts	12.5 (5.1)	12.5 (5.4)	0.974
Single object total inattention episode, counts	10.7 (5.4)	10.8 (5.4)	0.893
Multiple objects mean duration at toy, seconds	26.0 (18.3)	24.8 (13.2)	0.729
Multiple objects total duration at toy, seconds	222.3 (74.2)	239.9 (47.4)	0.195
Multiple objects total looks at toy, counts	10.9 (5.2)	11.9 (5.2)	0.411
Multiple objects total inattention episode, counts	8.1 (4.5)	8.7 (4.7)	0.578
Distractibility total duration TV/total duration toy	35.7 (28.8)	39.5 (24.3)	0.594
Distractibility latency, seconds	20.7 (10.4)	22.7 (18.3)	0.625
Distractibility total looks at TV, counts	11.1 (3.5)	11.3 (3.9)	0.824

Abbreviations: ARA, arachidonic acid; DHA, docosahexaenoic acid.Data presented as mean (SD). Between group comparisons by unadjusted general linear models.

Table S2. Major fatty acids levels of children at baseline and age 24 months

Fatty Acid	Baseline				Age 24 months		
	Supplement	Placebo	*p*		Supplement	Placebo	*p*
Palmitic acid				Palmitic acid			
RBC PE	11.6 (1.0)[2]	12.0 (0.8)	0.046	RBC PE	11.4 (0.8)	11.7 (0.8)	0.144
RBC PC	35.5 (1.7)	36.0 (1.6)	0.099	RBC PC	35.2 (1.8)	36.0 (2.4)	0.070
Plasma	30.5 (2.2)	30.3 (2.1)	0.599	Plasma	30.2 (2.1)	30.3 (1.2)	0.686
Stearic acid				Stearic acid			
RBC PE	5.68 (0.6)	5.76 (0.5)	0.479	RBC PE	5.88 (0.6)	5.83 (0.6)	0.652
RBC PC	13.5 (1.0)	13.2 (1.2)	0.242	RBC PC	13.7 (1.1)	13.0 (1.3)	0.010
Plasma	16.9 (1.3)	16.7 (1.3)	0.446	Plasma	16.8 (1.1)	16.6 (1.5)	0.326
Oleic acid				Oleic acid			
RBC PE	14.8 (1.1)	15.0 (1.3)	0.431	RBC PE	13.6 (1.0)	14.7 (1.1)	<0.001
RBC PC	16.1 (1.3)	16.0 (1.1)	0.568	RBC PC	15.6 (1.2)	15.7 (1.8)	0.734
Plasma	10.4 (1.3)	10.5 (1.3)	0.681	Plasma	9.48 (1.4)	10.4 (1.0)	<0.001

Abbreviations: PC, phosphatidylcholine; PE, phosphatidylethanolamine. Data presented as mean (SD). Between group differences determined by t-tests.
